# Regulation of *HOX* gene expression in AML

**DOI:** 10.1038/s41408-024-01004-y

**Published:** 2024-03-07

**Authors:** Irum Khan, Mohammed A. Amin, Elizabeth A. Eklund, Andrei L. Gartel

**Affiliations:** 1 Robert H. Lurie Comprehensive Cancer Center, Northwestern University, Chicago, IL USA; 2 Department of Medicine at the Feinberg School of Medicine, Northwestern University, Chicago, USA; 3 Jesse Brown VA Medical Center, Chicago, IL USA; 4 Department of Medicine, University of Illinois, Chicago, IL USA

**Keywords:** Oncogenes, Cancer epigenetics

## Abstract

As key developmental regulators, HOX cluster genes have varied and context-specific roles in normal and malignant hematopoiesis. A complex interaction of transcription factors, epigenetic regulators, long non-coding RNAs and chromatin structural changes orchestrate HOX expression in leukemia cells. In this review we summarize molecular mechanisms underlying HOX regulation in clinical subsets of AML, with a focus on NPM1 mutated (NPM1^mut^) AML comprising a third of all AML patients. While the leukemia initiating function of the NPM1 mutation is clearly dependent on HOX activity, the favorable treatment responses in these patients with upregulation of HOX cluster genes is a poorly understood paradoxical observation. Recent data confirm FOXM1 as a suppressor of HOX activity and a well-known binding partner of NPM suggesting that FOXM1 inactivation may mediate the effect of cytoplasmic NPM on HOX upregulation. Conversely the residual nuclear fraction of mutant NPM has also been recently shown to have chromatin modifying effects permissive to HOX expression. Recent identification of the menin-MLL interaction as a critical vulnerability of HOX-dependent AML has fueled the development of menin inhibitors that are clinically active in NPM1 and MLL rearranged AML despite inconsistent suppression of the HOX locus. Insights into context-specific regulation of HOX in AML may provide a solid foundation for targeting this common vulnerability across several major AML subtypes.

## Introduction

Acute myeloid leukemia (AML), an aggressive heterogenous hematological malignancy [1], is driven by recurrent cytogenetic abnormalities and hotspot mutations that suppress normal myeloid differentiation and prime malignant transformation [2–4]. AML is characterized by a hijacking of normal transcriptional networks [5]. The clustered homeobox (*HOX*) gene family of transcription factors have an established role in normal hematopoiesis and their deregulation has been linked to pathways critical for leukemic stem cell activity. Overexpression of several *HOX* family members in experimental models alters self-renewal and differentiation properties of hematopoietic stem and progenitor cells and increased levels of certain *HOX A* genes has been linked to unfavorable prognosis in patients with AML [6, 7].

The *HOX* genes contain a highly conserved nucleotide sequence called homeodomain and encode a large family of transcription factors. In humans, 39 *HOX* genes are organized into four genomic clusters with shared enhancers namely the *HOX A*, *B*, *C* and *D* clusters, located on chromosomes 7, 17, 12 and 2 respectively [8, 9]. These clusters allow for spatial and temporal regulation of transcription needed for development [3, 4]. *HOX A* and *HOX B* families, are critical for hematopoietic lineage development [10, 11]. While *HOX* 1–4 genes are highly expressed in stem and early hematopoietic progenitor cells, *HOX* 7–11 expression is maximal in lineage committee progenitors and there is progressive down regulation with terminal differentiation. The selective DNA binding of HOX proteins is heavily dependent on cofactors such as pre-B-cell leukemia (PBX) [12] and myeloid ecotropic insertion site (MEIS) families [13]. Given their fundamental developmental role, dysregulation of *HOX* genes through altered expression or mutation is strongly implicated in cancer biology [14]. Depending on cellular context, *HOX* genes can be proto-oncogenes or tumor suppressors. In the context of hematopoiesis [15, 16], induced overexpression of several *HOX* genes leads to progenitor cell expansion, differentiation arrest and in the case of *HOX A9* [17] and *A10* [18], development of AML.

**Fig. 1:**
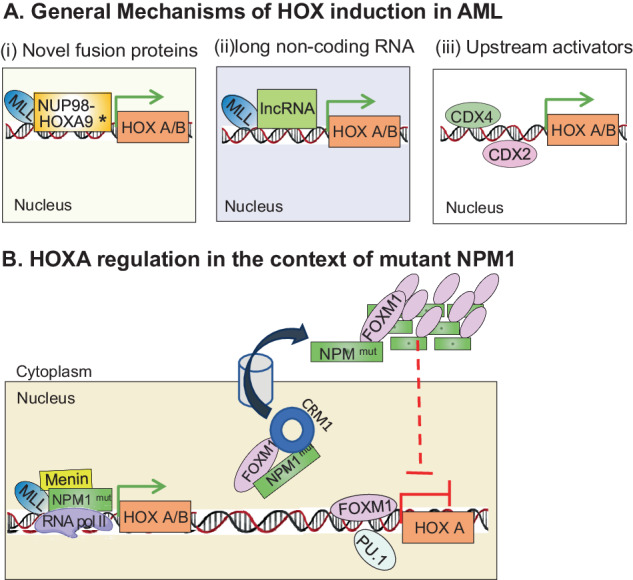
General and context-specific mechanisms of HOX regulation in AML. **A** Major mechanisms of regulation of expression of *HOX A/B* genes in human leukemia cells include (i) cytogenetic rearrangements resulting in novel fusion proteins, (ii) long non-coding RNA association with the HOX gene locus and (iii) binding of upstream protein such as CDX proteins. **B** Regulation of *HOX* A in the context of *NPM1*^mut^ AML includes two discrete mechanisms whereby (i) residual nuclear mutant NPM1 directly binds to chromatin and hijacks the transcriptional complex (RNA Pol II, MLL-Menin and super elongation complex) and (ii) the bulk of mutant NPM1 sequesters and relocalizes to the cytoplasm several repressors of HOX gene expression including FOXM1, and PU.1. * Additional cytogenetic arrangements inducing HOX expression include NUP98-NSD1, SET -NUP214, DEK-NUP214.

Numerous studies have shown that *HOX* genes can promote the development of AML by forming chimeric fusions with other genes [19, 20], or overexpression due to altered upstream regulators such as CDX proteins [21]. In this review we attempt to reconcile recent findings with known paradigms of *HOX* regulation focusing on HOX-dependent subsets of AML (Fig. 1).

### HOX regulation in *NPM1*^*mut*^ AML

Mutations of the nucleophosmin 1 (*NPM1*) gene are the most highly prevalent genomic alteration present in nearly a third of adult AML patients [22–27]. NPM1, a ubiquitously expressed protein while mainly located in the nucleolus, continuously shuttles between nucleus and cytoplasm. [28, 29]. It is involved in multiple cellular functions including histone assembly, ribosome biogenesis and export, centrosome duplication, maintenance of genome stability, transcriptional regulation, and DNA repair [30]. *NPM1* mutations are always heterozygous and mainly restricted to exon 12 [24, 31, 32] and result in the insertion of a new C-terminal nuclear export signal on the protein [24] whereby mutated NPM heterodimerizes with wild type NPM and is translocated from the nucleus to cytoplasm with the help of nuclear exporter CRM1 [33–35]. *HOX* gene (specially *HOXA*/*B*) upregulation is a pathognomonic feature of *NPM1*^*mut*^ AML [15, 36–41] and a key consequence of this mutation. Nuclear relocalization of the mutant NPM protein by inactivating the nuclear export signal (NES) or targeted deletion *of NPM1*^*mut*^ by CRISPR-Cas9 genome editing technology results in rapid loss of *HOX* gene expression, abrogates the undifferentiated state and induces growth arrest [42, 43]. Thus, NPM acts as a direct upstream activation factor of *HOX* gene expression to maintain the growth and undifferentiated state of *NPM1*^*mut*^ AML cells. The mechanism underlying *NPM1*^*mut*^ regulation of *HOX* has been the subject of many recent studies.

A recently described mechanism links mutant *NPM1* to the regulation of the *HOX A/B* gene clusters and the oncogenic state of AML cells via cytoplasmic export of the Forkhead box M1 (FOXM1) transcription factor. FOXM1, a member of the Forkhead family transcription factors, promotes cell proliferation, survival, tissue invasion, metastasis, angiogenesis, therapy resistance, and stem cell self-renewal [44–50]. Its overexpression is strong predictor of poor outcomes across many cancers including AML [45, 49, 51, 52]. Wild-type NPM binds FOXM1 through the heterodimerization domain of NPM (residues 187–259) and regulates its intracellular localization and stability [53]. This interaction was confirmed in AML cell lines and primary samples with mutant and wild type *NPM1* [51]. In AML cells expressing *NPM1*^*mut*^, FOXM1 is inactivated and localized in the cytoplasm together with mutant cytoplasmic NPM (NPM1c). Stable knockdown of FOXM1 in AML cell lines expressing wild-type *NPM1* resulted in significant upregulation of *HOX A* cluster genes in RNA-seq analysis recapitulating the phenotype of *NPM1*^*mut*^ cells [54] and conferred sensitivity to current therapies including cytarabine and venetoclax. Therefore, FOXM1 may be a key intermediate in the *NPM1*^*mut*^ */ HOX* axis whereby transcriptional inactivation of FOXM1 by cytoplasmic sequestration allows de-repression of *HOX* cluster genes. Haploinsufficiency of *FOXM1* was recently shown to accelerate leukemogenesis by enhancing self-renewal in short term HSC [55] and DNA-damage, although a downstream effect on HOX expression was not examined. A role for FOXM1 as a tumor suppressor was demonstrated in a breast cancer model where FOXM1 was shown to interact with Rb and caused transcriptional suppression of the mammary alveolar differentiation program [56]. Recent RNA-seq analysis from the BEAT AML study showed that FOXM1 is transcriptionally downregulated and several *HOX* genes are significantly upregulated in *NPM1*^*mut*^ AML patients [57] strengthening the evidence for negative correlation of FOXM1 transcriptional activity and HOX expression in primary samples. RNA inhibition of FOXM1 in AML cells resulted in increased expression of *HOXA* genes suggesting FOXM1 is sufficient to regulate HOX expression, independent of NPM molecular status [54]. These data suggest that the oncogenicity and chemosensitivity of NPM1 mutant AML cells can be simultaneously reconciled by transcriptional inactivation of FOXM1 and consequent de-repression of *HOX A/B* cluster genes.

Additional nuclear binding partners of NPM including myeloid transcription factor PU.1 and the architectural protein CCCTC binding factor CTCF, and both are required for differentiation and physiological *HOX* downregulation. Mutant NPM directly binds and translocates these putative factors from the nucleus to cytoplasm [58, 59]. The self-renewal and undifferentiation phenotype of PU.1-null myeloid precursor is significantly abrogated by reintroduction of nuclear PU.1 [58]. Deletion of CTCF in AML cells induced *HOXA9* gene expression [60]. The data obtained from these investigations suggest that mutant nucleophosmin acts as a derepressor of *HOX* gene expression indirectly by translocating *HOX* repressors such as PU.1 and CTCF from the nucleus to cytoplasm. These data collectively support the premise that cytoplasmic relocalization of NPM and its binding partners is critical to the oncogenic function of this mutation.

Previous studies reported that mutant *NPM1* could directly regulate *HOX* gene expression through an unknown but potentially chromatin-associated mechanism [42, 61, 62]. Recent studies elegantly demonstrated that mutant nucleophosmin (NPM1c) binds a subset of gene promoters and transcriptionally upregulates HOXA/B cluster genes through distinct mechanisms. The mutant NPM1c protein was shown to bind to chromatin at MLL-menin binding sites amplifying the function of this histone methyltransferase [63] while also inhibiting the activity of histone deacetylases [64] to maintain active transcription This chromatin bound mutant NPM directly modulates the active nascent transcription of *HOXA/B* cluster genes and cofactor MEIS1 by controlling the overall transcriptional activity mediated by transcriptional complexes such as RNA polymerase II, MLL-Menin complex and super elongation complex [63, 64].

Long non-coding RNAs (lncRNAs), a major transcribed product of the genome, play a role in chromatin remodeling or directing transcription factors to their target genomic sequences It was recently shown that lncRNA HOTTIP drives aberrant posterior *HOXA* gene expression through alteration of topologically associated domain (TADs) in the AML genome without affecting the anterior *HOXA* locus [65]. HOTTIP lncRNA is aberrantly expressed in the subset of AML patients with *NPM1*^*mut*^ and *MLL* rearrangements [65] where it regulates the recruitment of WDR5/MLL complex to coordinate active chromatin modifications and HOXA gene expression [66]. Loss of HOTTIP lncRNA leads to reduced leukemic burden in an *MLL* rearranged mouse model and leukemogenesis by *HOX A* TADs is restored by reactivation of HOTTIP.

A more recent study [67] introduced the novel concept of long non coding RNA from the HOX B locus-HOXBLINC as a critical downstream mediator of NPM1c + -associated leukemic transcription program and leukemogenesis.

In summary, we describe three complimentary mechanisms convergently regulating the *HOX A/B* locus in NPM1 mutant AML, namely (i) cytoplasmic delocalization of repressors, (ii) chromatin modification by NPM1c directly or (iii) indirectly through long non coding RNA. Collectively this data underscores the critical oncogenic dependency on HOX A/B expression in this genomic subset of AML. Earlier work has shown that high HOX expression maintains the leukemic state in NPM1-mutant AML [42] and we postulate this results in potentially redundant mechanisms to sustain expression of this key transcription factor family.

### HOX regulation in AML with recurrent cytogenetic abnormalities

One of the earliest defined subsets of AML is characterized by chromosomal translocations which generate leukomogenic fusion proteins that often act as aberrant transcription factors [68]. Recurrent chromosomal translocations in AML influencing HOX include direct fusion of *HOX* with nucleoporin genes such as *NUP98* and rearrangements that involve upstream regulators, such as the mixed lineage leukemia gene (*MLL*) [69, 70].

#### NUP98

It has been reported that the recurring translocations in AML fuse *NUP98* directly to *HOXA9* and *HOXD13*, as well as to histone methyltransferase nuclear receptor-binding SET domain protein 1 (*NSD1*) [71–76]. NUP98-NSD1 fusion protein has been reported to induce AML in vivo, maintain self-renewal of myeloid stem cells in vitro and enforce expression of proto-oncogenes such as *HOXA* (*A7, A9 and A10*) and *MEIS1*. NUP98-NSD1 directly activates *HOXA* gene expression by binding to chromatin regions marked by histone H3 Lys 36 methylation (H3K36me) and prevents transcriptional repression of *HOXA* locus genes mediated by binding of EZH2-complexes to the *HOXA* gene locus [77]. A study using mouse embryonic stem cells showed that NUP89-HOX9A is significantly recruited to the *HOX* cluster region with the help of CRM1 and activates expression of *HOX* genes [78]. NUP98-homebox fusions have greater effect on self-renewal and aberrant gene expression than NUP98-nonhomebox fusions [79]. NUP98 fusion proteins such as NUP98-HOXA9, NUP98-HOXD13, and NUP98-NSD1 interact with non-specific lethal (NSL) and MLL1 histone-modifying complexes to drive HOX gene expression [75]. Analysis of chromatin immunoprecipitation sequencing data showed that NUP98-HOXA9 and MLL1 are recruited to *HOXA* and *HOXB* cluster gene loci marked by H3K4me3 and H4K16ac. Inactivation of MLL1 significantly reduces NUP98-HOXA9-induced gene expression as well as growth and survival of NUP98-HOXA9 driven leukemogenesis in vitro and in vivo [75]. These results demonstrate that NUP98 fusion protein mediates leukemogenesis and elevated HOX gene expression by controlling the gene transcriptional activity of histone modifying complex. In addition to direct transcriptional activation through the homeodomain, the NUP98 moiety also plays an important role in transformation by inhibiting CRM1-mediated nuclear export with resultant retention and enhanced activity of oncogenic transcription factors NFAT and NFκB [80].

### NUP214

NUP214 is another nucleoporin involved in translocations with two chromatin remodeling proteins SET and DEK resulting in fusion proteins that influence *HOX* expression [70, 74, 76, 81, 82]. SET-NUP214 is associated with both acute lymphoblastic leukemia (ALL) as well as AML, while DEK-NUP214 is exclusively involved in AML. The SET-NUP214 fusion protein is recruited to the *HOX* cluster region with help of CRM1 where it recruits active RNA polymerase II leading to aberrant transcriptional activity of HOX genes [78]. DEK-NUP214 fusion protein also causes upregulation of *HOX* genes, specially *HOXA9* in AML [83].

#### MLL

It has previously been reported that histone methyltransferase *KMT2A* (*MLL1*- mixed-lineage leukemia1) is genetically rearranged in upto 10% of AML with more than 64 translocation partner genes [84] and transformation of murine bone marrow by MLL fusion proteins is *Hox* gene dependent [85]. MLL directly binds to and utilizes its highly conserved SET domain H3 (Lys4) methyltransferase to regulate HOX promoters. Leukemia associated MLL-fusion proteins have deletion of the SET domain that may compromise stage-specific down regulation of *HOX* transcription [86] .

### Upstream mechanisms of HOX regulation in AML

#### CDX

The *CDX* gene, a member of the ParaHox gene family has been shown to influence organogenesis and hematopoiesis through regulation of *HOX* gene expression and Cdx proteins can act directly on *Hox* gene regulatory elements [87, 88]. CDX2 is aberrantly expressed in leukemic cells of 90% AML patients [21]. Three CDX homologs CDX1, CDX2, and CDX4 have consensus binding sites in the promoters of multiple *HOX* genes [87]. Both in vivo and in vitro experiments showed that *CDX2* and *CDX4* expression led to dysregulation of *HOX* expression. Overexpression of *CDX2* leads to aberrant *HOX* gene expression in murine hematopoietic progenitors and this link was confirmed in AML primary cells [21, 89]. CDX2 acts as a positive upstream regulator of several *HOX* genes such as *HOXA6/10* and *HOXB8,* and the proliferation of AML cell lines was inhibited by siRNA targeting of CDX2 [89].

In addition, overexpression of CDX4 can transform hematopoietic cells in cooperation with MEIS1 leading to AML in mouse models [90]. CDX4 and HOXA10 were shown to regulate one in a positive feedback loop [91], with the HOXA10 promoter having a CDX4-binding cis element and vice versa. β-catenin also activates CDX4 transcription and is upregulated as a consequence of HOX effects on FGF2 [92, 93]. Thus, there is cross regulation at several levels between HOX, HOX target genes such as FGF2, β-catenin and CDX.

Our review highlights a confluence of different mechanisms regulating HOX expression. Hox-binding cis elements often occur in tandem in genes (like the CYBB, NCF2 and ARIH2 genes) [94] with differential binding affinity for each of the “cassettes” that bind Hox proteins. This tremendous flexibility in the regulation of target gene expression is germane to the developmental role of HOX transcription factors.

### Challenges in targeting HOX in leukemia and future directions

Using a CRISPR/Cas9 genome editing screen [95], the histone modifier MLL1 was shown to be critical factor regulating HOX expression in NPM1^mut^ AML and the menin binding site of MLL was proven to be a potent target in NPM1^mut^ AML. Early therapeutic development focused on DOT1L, the histone methyltransferase that specifically targets nucleosomal H3K79 aberrantly recruited to MLL target genes including HOXA9 but these inhibitors lacked specificity and clinical trials of EPZ-5676 showed variable effects on histone methylation and HOXA9 inhibition not necessarily correlated with clinical outcomes [96]. Recent therapeutic development of menin inhibitors in HOX-addicted AML has been very encouraging leading to ongoing review by the FDA for a first-in-class menin inhibitor in AML [97]. However the inhibitory activity of these compounds is predominantly at the co-factors MEIS and PBX3 with inconsistent suppression of the HOXA locus [98]

Context-specific knowledge of transcriptional regulation of *HOX* expression has given impetus to therapeutic targeting in genomic subsets of AML. In addition, recent data on direct effects of mutant NPM1c on chromatin structure may uncover novel targets. There is increasing interest in cross regulation of HOX proteins by each other and other homeodomain proteins such as CDXs as well as potential targets downstream of HOX such as FGF2. There is an ongoing clinical trial with nintedanib (FGFR inhibitor) in HOXA overexpressing leukemia (NCT03513484).

While enhanced oncogenic activity is a functional property of HOX overexpression that is being aggressively targeted, the relationship of HOX overexpression to increased sensitivity to chemotherapy and bcl2 inhibitors [37, 99] remains elusive. It is still to be determined whether high HOXA expression in chemosensitive subsets of AML indicates an alternate functional role of HOXA as an effector of treatment responses or a biomarker for loss of FOXM1 or additional mediators of chemoresistance.
